# Comparative Genomic Analysis of *Vibrio cincinnatiensis* Provides Insights into Genetic Diversity, Evolutionary Dynamics, and Pathogenic Traits of the Species

**DOI:** 10.3390/ijms23094520

**Published:** 2022-04-20

**Authors:** Yuhui Du, Yuan Jin, Beiping Li, Junjie Yue, Zhiqiu Yin

**Affiliations:** 1 MOE International Joint Research Laboratory on Synthetic Biology and Medicines, School of Biology and Biological Engineering, South China University of Technology, Guangzhou 510000, China; duyuhui@scut.edu.cn; 2 Laboratory of Genetic Engineering, Beijing Institute of Biotechnology, Beijing 100000, China; jinyuan@bmi.ac.cn (Y.J.); libp@bmi.ac.cn (B.L.); 3 National Engineering Research Center for Efficient Utilization of Soil and Fertilizer Resources, College of Resources and Environment, Shandong Agricultural University, Tai’an 271000, China

**Keywords:** *Vibrio cincinnatiensis*, pan-genome, mobile genetic elements, horizontal gene transfer, virulence

## Abstract

*Vibrio cincinnatiensis* is a poorly understood pathogenic *Vibrio* species, and the underlying mechanisms of its genetic diversity, genomic plasticity, evolutionary dynamics, and pathogenicity have not yet been comprehensively investigated. Here, a comparative genomic analysis of *V. cincinnatiensis* was constructed. The open pan-genome with a flexible gene repertoire exhibited genetic diversity. The genomic plasticity and stability were characterized by the determinations of diverse mobile genetic elements (MGEs) and barriers to horizontal gene transfer (HGT), respectively. Evolutionary divergences were exhibited by the difference in functional enrichment and selective pressure between the different components of the pan-genome. The evolution on the Chr I and Chr II core genomes was mainly driven by purifying selection. Predicted essential genes in *V. cincinnatiensis* were mainly found in the core gene families on Chr I and were subject to stronger evolutionary constraints. We identified diverse virulence-related elements, including the gene clusters involved in encoding flagella, secretion systems, several pili, and scattered virulence genes. Our results indicated the pathogenic potential of *V. cincinnatiensis* and highlighted that HGT events from other *Vibrio* species promoted pathogenicity. This pan-genome study provides comprehensive insights into this poorly understood species from the genomic perspective.

## 1. Introduction

*Vibrio* is a genus of ubiquitous bacteria found to be widely distributed in aquatic and marine ecosystems worldwide [1]. The genus *Vibrio* contains over 100 described species, a dozen of which cause infections in humans and aquatic animals. Non-cholera *Vibrio* species, such as well-known *V. vulnificus*, *V. parahaemolyticus*, and *V. alginolyticus*, cause vibriosis infections, including gastroenteritis, soft-tissue infections, septicemia, and meningitis [2]. These infections are usually associated with foreign travel, seawater exposure, and contaminated food ingestion. Most *Vibrio* species share several biological and genomic features, including two differently sized chromosomes, genetic diversity associated with mobile genetic elements (MGEs), recombination, and horizontal gene transfer (HGT) [3,4]. Comparative genomic analysis of less-studied *Vibrio* species allows researchers to investigate fundamental questions regarding the genetic diversity, pathogenic mechanisms, evolutionary relationships, and genomic dynamics within the *Vibrio* genus.

*V. cincinnatiensis*, represented as a species in the genus *Vibrio*, is a halophilic Gram-negative bacterium [5]. *V. cincinnatiensis* was first isolated from a 70-year-old male at the University of Cincinnati Hospital in 1986 as an infectious agent of septicemia and meningitis [6]. Then, in 1993, it was reported to be isolated from a diarrheic immunocompromised patient [7]. Furthermore, *V. cincinnatiensis* was found to be associated with antimicrobial resistance and harbor several resistance-related genes, including *sul1*, *sul2*, *aadA2*, and the class 1 integron [8]. It also appeared resistant to the complement-mediated lysis of humoral fluids of amphioxus [9]. *V. cincinnatiensis* as veterinary isolates with zoonotic potential have also been reported. In China, as one of the bacterial pathogens, it caused mud crab mortalities and serious economic losses [10]. Recently, Jäckel et al. collected more than 100 suspected veterinary *V. cincinnatiensis* isolates from the veterinary laboratory at Dresden over a period of 20 years [11]. Bai et al. determinated several virulence-related genes in *V. cincinnatiensis* using PCR amplification, including haemolysin, *toxS*, *flaB,* and *flaC* [12]. However, the underlying mechanisms of pathogenicity need to be comprehensively investigated from a whole-genome perspective.

*V. cincinnatiensis* has been reported to be distributed in aquatic environments worldwide [13]. It was found in water samples collected from the Choptank River in Chesapeake Bay of USA [13]. The occurrence of *V. cincinnatiensis* in mussels harvested from Adriatic Sea (Italy) was reported [14]. In addition, *V. cincinnatiensis* was also isolated from the freshwater environment of Hiroshima (Japan) and Lima (Peru) [15,16]. Global marine habitat is the ecological niche of many microorganisms, which promote genetic diversity by the acquisition of genetic material by HGT from other organisms [3]. Thus, the genetic diversity, mobile-related genetic elements, and potential role of HGT in the *V. cincinnatiensis* pan-genome deserve comprehensive research.

Until now, due to limited studies that focus on *V. cincinnatiensis*, very little molecular data have been presented. Comparative analyses by whole-genome data could offer tremendous advantages for the evaluation of genetic diversity and phylogenetic relationship and determining MGEs, virulence-related elements, and resistance genes [17,18,19,20]. Presently, there are six available genomes of *V. cincinnatiensis* in National Centre for Biotechnology Information (NCBI) GenBank up to 30 January 2022. In this study, we constructed a pan-genome analysis of the species *V. cincinnatiensis* to evaluate the genetic diversity. Then, the genomic plasticity was evaluated by the analysis of MGEs and barriers to HGT. Comparison analyses were performed to investigate the evolutionary divergence in biological function and selective pressure between each component of the pan-genome and each chromosome. Phylogenetic analysis was constructed to explore the phylogenetic position of *V. cincinnatiensis*. Genetic properties (e.g., fimbrial operon, secretion system operon, and virulence genes) that were observed in the pan-genome revealed the underlying pathogenic mechanisms of *V. cincinnatiensis*.

## 2. Results and Discussion

### 2.1. Genomic Information for V. cincinnatiensis

All available sequenced *V. cincinnatiensis* genomes were collected from the NCBI GenBank database (Table S1; (accessed on 30 January 2022)). The collection contained six genomes, of which four genomes were complete. These genomes were estimated to be of 98.7 ± 0.2% completeness with 1.9 ± 0.1% contamination (Table S1). Then, gene prediction and annotation were performed using a consensus approach based on RAST server. The general characteristics of the six genomes of *V. cincinnatiensis* are shown in Table 1. The average genome size was 3.8 ± 0.05 Mb with 43.8 ± 0.04% GC-content and 3509 ± 40 RAST-predicted protein-coding genes. The estimated size of chromosome I (Chr I) averaged 2.8 ± 0.04 Mb with 44.2 ± 0.07% GC-content and 2554 ± 24 genes, whereas, for chromosome II (Chr II), the estimated size averaged 1.007 ± 0.02 Mb with 42.6 ± 0.1% GC-content and 955 ± 35 genes.

### 2.2. Pan-Genome Analyses Revealed That a High Degree of Genetic Variability on Chr II Promoted the Genetic Diversity of V. cincinnatiensis

**Figure 1:**
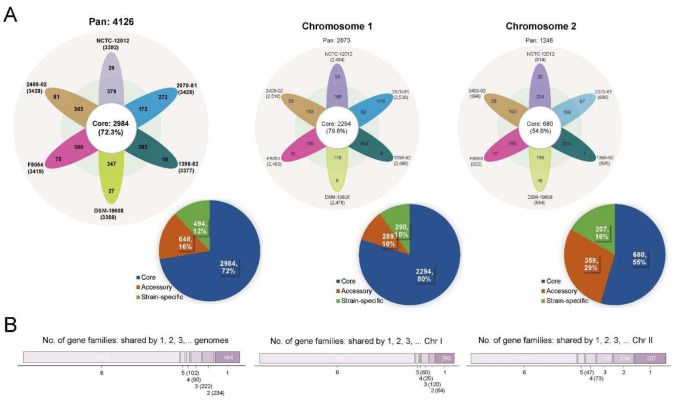
The pan-genome of the *V. cincinnatiensis* genome, Chr I, and Chr II. (**A**) Flower plots represent the number of the core, accessory, and strain-specific gene families shared by each strain. Pie charts represent the percentage of the core, accessory, and strain-specific gene families participating in the pan-genome of the *V. cincinnatiensis* genome, Chr I, and Chr II, respectively; (**B**) distribution of the gene families in the pan-genome of the *V. cincinnatiensis* genome, Chr I, and Chr II.

We characterized the pan-genome among six *V. cincinnatiensis* genomes to assess their genetic diversity. A total of 4126 gene families were identified (Figure 1A and Table S2). Among these, 2984 (72.3%) gene families represented the core genome and the remaining 1142 (27.7%) variable gene contents consisted of the accessory genome (648; 15.7%) and strain-specific genes (494; 12.0%). As shown in Figure 1B, most variable genes were not broadly distributed. The size of the accessory genome in *V. cincinnatiensis* genomes ranged from 172 to 383 gene families with an average of 334.0 ± 80.4 gene families. The size of the strain-specific gene contents ranged from 10 to 272 genes with an average of 82.3 ± 97 genes. Thus, the composition of each genome, concerning the variable gene contents, is remarkably divergent. In addition, we identified 31 gene families shared by both chromosomes, which included 17 core gene families and 14 accessory gene families.

In previous papers, Chibani et al., constructed the pan-genome analysis of 15 *Vibrio alginolyticus* genomes and found that the core genome (3876 gene families) becomes smaller than the accessory gene pool (4967 gene families) [21]. Meanwhile, Nathamuni et al., revealed that the pan-genome of seven *Vibrio* species (35 genomes) consisted of 17,033 genes, which included 2004 (11.8%) core, 8249 (48.4%) accessory, and 6780 (39.8%) unique genes, respectively [22]. Thus, our results might represent a smaller genetic diversity than the actual value due to the limitation of only six genomes. This is further limited by strains NCTC-12012 and DSM-19608 being the same type strain while strain 1398-82 is highly similar to both (99.99% ANI) and may be the same strain. A comprehensive dataset, which represents an even distribution of *V. cincinnatiensis* genomic diversity, would be required in future work.

With respect to the chromosomes, the analysis of the pan-genome resulted in 2873 gene families for Chr I and 1246 gene families for Chr II (Figure 1A). Notably, the core pan-genome for Chr I made up 79.8% (2294 gene families) of the Chr I pan-genome, whilst only 55.4% (690 gene families) was core pan-genome for the Chr II pan-genome. The accessory pan-genomes were 289 and 359 gene families for Chr I and Chr II, respectively. The strain-specific gene families for Chr I and Chr II were 290 and 207, respectively. The Chr I pan-genome is larger and more stable, whereas the Chr II pan-genome is smaller and more variable. The Chr II pan-genome accounts for more of the genetic variability of *V. cincinnatiensis*. The comparison of both chromosomes is shown in Figure 1.

### 2.3. An Open Pan-Genome of V. cincinnatiensis Exhibited the Potential for Genetic Innovation, Especially for Chr II

**Figure 2:**
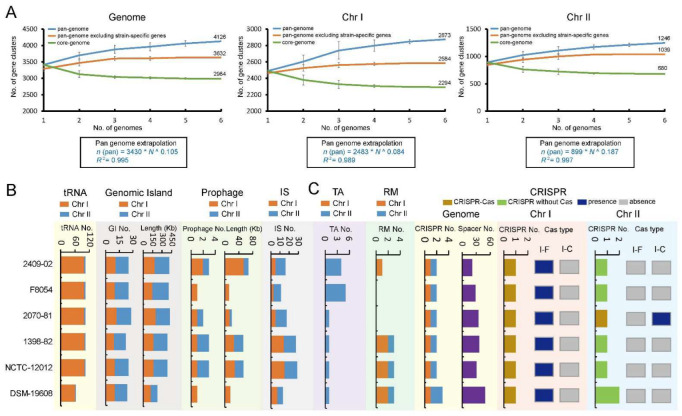
(**A**) The progressive curves for the core, pan-genome, and pan-genome excluding strain-specific genes of the *V. cincinnatiensis* genome, Chr I, and Chr II. The curves showed the downward trend of the core gene families and the upward trend of the pan-gene families with additional genomes. The deduced mathematical functions of pan-genome curves were shown below the curves; (**B**) distribution of mobile genetic elements; (**C**) distribution of multiple barriers to horizontal gene transfer.

The *V. cincinnatiensis* pan-genome growth curves showed a clear linear progression fitting Heap’s law (*n* = $кN^{\gamma }$) pan-genome model [23] (Figure 2A). The pan-genome size steadily increased with the addition of each additional genome, with ~119 new genes added on average to the pan-genome as each new *V. cincinnatiensis* genome was added. The growth exponent values of parameter *γ* were 0.1 for the genome versus 0.08 for Chr I and 0.2 for Chr II, which were above the critical threshold *γ* = 0 [23], indicating an open pan-genome. Similarly, some aquatic bacteria, such as *Aeromonas* and *Plesiomonas shigelloides*, also have open pan-genomes and represent ecological adaptation with genetic diversity [24,25]. However, it is notable that when strain-specific genes are excluded from a pan-genome, a plateau in the pan-genome curves results, indicating that most undiscovered genes are likely not broadly distributed. Moreover, the upward trend and the growth exponent value of the Chr II pan-genome curve are greater than that of the Chr I pan-genome curve (Figure 2A), suggesting Chr II may have more frequent genetic exchange events. In contrast to the increase in the size of the pan-genome, the core genome curves decreased with additional genomes, reaching minimum values of 2984, 2294, and 880 for genome, Chr I, and Chr II, respectively.

### 2.4. Distribution of tRNAs, Mobile Genetic Elements, and Multiple Barriers in the V. cincinnatiensis Genomes

MGEs can mediate the transmission of genetic material and facilitate the expansion of gene pools of bacterial taxa [26]. Notably, MGEs facilitate the acquisition of genes conferring pathogenicity and antibiotic resistance during the emergence of pathogenicity [27,28]. In the *V. cincinnatiensis* genomes, we identified the tRNA loci and multiple types of MGEs, including genomic islands (GIs), prophages, and insertion sequences (ISs) (Table S3). On average, one genome contained 96.8 ± 16.1 tRNAs, 24.8 ± 1.9 GIs (370.3 ± 73.4 kb in size), 2.2 ± 1.0 prophages (36.9 ± 24.2 kb in size), and 18.8 ± 7.4 ISs. As shown in Figure 2B, numerous MGEs are heterogeneously distributed in the *V. cincinnatiensis* genomes. Most tRNA loci were located on Chr I. GIs spanned almost 10% of the genome. The number (14.5 ± 1.0) and covering regions (205.3 ± 52.0 kb) of GIs for Chr II were more than Chr I (10.3 ± 1.0; 165.0 ± 28.7 kb). The distribution of prophages and ISs presents individual differences. For example, 2409-02 genome harbored three prophages sized 54.8 kb, whereas F8054 genome had only one prophage sized 11.8 kb. Similarly, 29 ISs were located in NCTC-12012 genome, and only 11 ISs were present in F8054. Similarly, Chibani et al., demonstrated in *V. alginolyticus* isolates that the formation of ecotypes and speciation is mainly driven by HGT through MGEs [21]. Thus, the existence of diverse MGEs contributed to the expansion of the open pan-genome of *V. cincinnatiensis*.

Several genetic elements as barriers to HGT have been proposed that control genome stability in bacteria [29]. These elements defend microbes against recurrent bacteriophage and plasmid infection and prevent foreign DNA uptake [30]. In this study, we also identified multiple types of barriers in the *V. cincinnatiensis* genomes, including restriction-modification (RM) system, toxin/antitoxin (TA) systems, and clustered repetitively interspaced palindromic repeat (CRISPR) (Figure 2C). Interestingly, all identified TA operons were heterogeneously distributed on Chr II; 2409-02 and F84054 Chr II harbored four and five TA operons, respectively, and the remaining strains contained only one TA operon. In contrast, most identified RM operons were present on Chr I; F84054 and 2070-81 genomes have no RM operons. Furthermore, we also identified CRISPR-Cas loci and CRISPR (without Cas protein) loci in *V. cincinnatiensis*. The number of the spacers in these CRISPR loci is variable. The type I-F CRISPR-Cas system was located on Chr I of all genomes, and type I-C CRISPR-Cas system was only present on Chr II of the 2070-81 genome. The existence of these barriers with HGT might play an important role in the genome stability of *V. cincinnatiensis*.

### 2.5. Comparison of Functional Characteristics for Pan-Genome Revealed the Evolutionary Divergence

**Figure 3:**
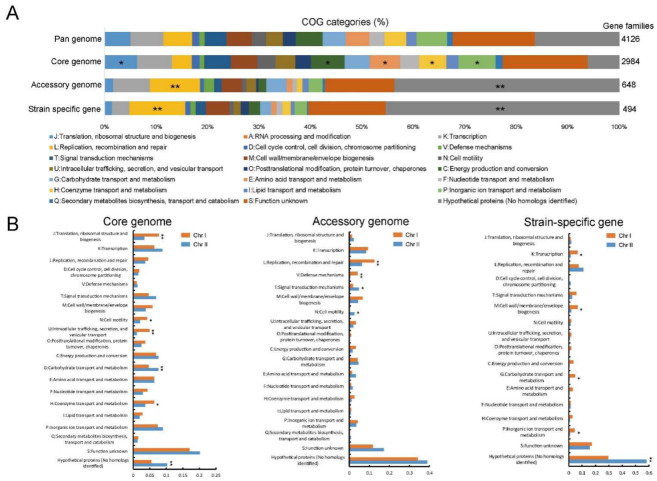
Comparison of functional enrichment for pan-genome. (**A**) Distribution of clusters of orthologous groups (COG) functional categories for pan-genome, core genome, accessory genome, and strain-specific genes, respectively. *: Fisher’s exact test *p*-value < 0.05; **: Fisher’s exact test *p*-value < 0.01; (**B**) comparison of COG functional categories for pan-genome, core genome, accessory genome, and strain-specific genes between Chr I and Chr II, respectively. *: *t*-test *p*-value < 0.05; **: *t*-test *p*-value < 0.01.

To gain insight into the functional characteristics of the pan-genome, we categorized the functions of the core, accessory, and strain-specific gene families using the clusters of orthologous groups (COG) assignments. The gene families not annotated with COG functional categories were defined as “hypothetical proteins”. The core gene families were significantly associated with metabolism categories (e.g., “C: energy production and conversion”, “E: amino acid transport and metabolism”, “H: coenzyme transport and metabolism”, and “P: inorganic ion transport and metabolism”) and “J: translation, ribosomal structure and biogenesis” (isher’s exact test *p* value < 0.05), indicating the conservation in metabolic functions (Figure 3A). Gene families involved in “L: replication, recombination and repair” and “hypothetical proteins” (Fisher’s exact test *p* value < 0.01) were significantly enriched in the accessory genome and strain-specific gene content (Figure 3A), indicating the presence of potential HGT events promoted the genetic diversity of the *V. cincinnatiensis* pan-genome.

The difference of genetic variability occurring at the chromosome level was observed earlier in the pan-genome analysis. Here, to explore the potential functional difference between Chr I and Chr II, we compared the functional categories of the core, accessory, and strain-specific gene families at the chromosome level. As shown in Figure 3B, significant differences in functional categories were observed in different components of the pan-genome. In the core genome, Chr I harbored higher percentages for gene families involved in “J: translation, ribosomal structure, and biogenesis”, “U: intracellular trafficking, secretion, and vesicular transport” (*t*-test, *p* < 0.01), “N: cell motility”, and “H: coenzyme transport and metabolism” (*t*-test, *p* < 0.05), whereas the core gene families of Chr II were enriched in “G: carbohydrate transport and metabolism” and “hypothetical proteins” (*t*-test, *p* < 0.01). Interestingly, the Chr I accessory gene families were significantly associated with “L: replication, recombination, and repair” and “V: defense mechanisms” (*t*-test, *p* < 0.01) (Figure 3B), highlighting that Chr I also was closely related to HGT events and their barriers despite the lack of genetic variability. Meanwhile, gene families assigned to “T: signal transduction mechanisms” and “N: cell motility” were prominently represented in the Chr II accessory genome (*t*-test, *p* < 0.05). The Chr I strain-specific genes were significantly responsible for “K: transcription”, “M: cell wall/membrane/envelope biogenesis”, “G: carbohydrate transport and metabolism”, and “P: inorganic ion transport and metabolism” and were enriched on Chr I (*t*-test, *p* < 0.05). Significantly, the Chr II had a high proportion of strain-specific genes with “hypothetical proteins” (*t*-test, *p* < 0.01). These unknown functional genes with limited distribution contributed to the Chr II genetic diversity and their biological roles require further research.

### 2.6. Selective Pressure Analysis Exhibited the Dynamics of Natural Selection in Core Genome

**Figure 4:**
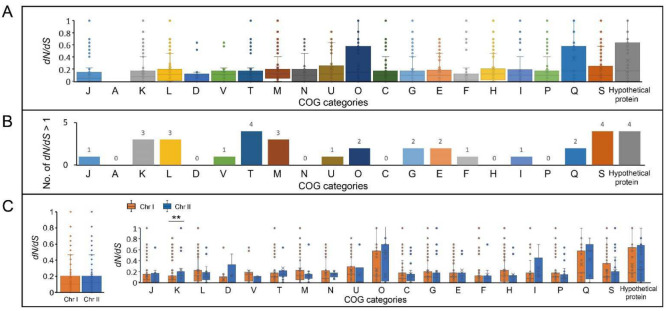
Selective pressure on functional categories in the *V. cincinnatiensis* core genome. (**A**) Distribution of the nonsynonymous (*dN*)/synonymous (*dS*) rates of the core gene families in COG functional categories; (**B**) distribution of COG categories for gene families with positively selected sites; (**C**) comparisons of the *dN*/*dS* rates of core gene families between Chr I and Chr II in COG functional categories. ** *t*-test *p*-value < 0.01.

To explore how natural selection shapes the *V. cincinnatiensis* genetic properties, we performed a codon-level analysis of natural selection on the 2529 mutational core gene families that represented many housekeeping functions for *V. cincinnatiensis*. Signatures of the evolution of these core gene families were measured by the nonsynonymous/synonymous rate ratio (*dN*/*dS*). The *dN/dS* rates of most of the core gene families (*n* = 2459, 97.2%; average *dN*/*dS* = 0.2 ± 0.3) were less than 1, exhibiting a predominant action of purifying selection in the *V. cincinnatiensis* core genome. These remarkable evolutionary constraints might be due to the functional importance of these core gene families, and they would maintain a stable and adapted genomic core. We further investigated the evolutionary dynamics in each functional category. Gene families assigned to “D: cell cycle control, cell division, and chromosome partitioning” (average *dN/dS* = 0.07 ± 0.1), “F: nucleotide transport and metabolism” (average *dN/dS* = 0.1 ± 0.2), and “J: translation, ribosomal structure, and biogenesis” (average *dN/dS* = 0.1 ± 0.2) exhibited stronger evolutionary constraints (Figure 4A). By contrast, genes related to “Q: secondary metabolites biosynthesis, transport, and catabolism” (average *dN/dS* = 0.4 ± 0.4), “hypothetical proteins” (average *dN/dS* = 0.4 ± 0.4), and “O: posttranslational modification, protein turnover, and chaperones” (average *dN*/*dS* = 0.3 ± 0.3) underwent weaker evolutionary constraints (Figure 4A). The results indicated that different functional categories have undergone different degrees of evolutionary constraints, indicating that different evolutionary strategies operated on the *V. cincinnatiensis* core genome. A total of 34 core gene families were identified as positively selected (*dN/dS* > 1) (Table S4). These gene families undergoing positive selection were involved in several functional categories, such as “S: function unknown”, “hypothetical proteins”, and “T: signal transduction mechanisms” (Figure 4B).

We also compared the evolutionary signatures of the core genome between Chr I and Chr II. Overall, the Chr I core gene families (average *dN*/*dS* = 0.2 ± 0.3) experienced a similar degree of selective pressure as the Chr II core gene families (average *dN*/*dS* = 0.2 ± 0.3) (Figure 4C). Furthermore, the similar degree of evolutionary pressure was also observed in the majority of functional categories between Chr I and Chr II, except for “K: Transcription” (Chr I: average *dN*/*dS* = 0.1 ± 0.2; Chr II: average *dN*/*dS* = 0.3 ± 0.3; (-test, *p* < 0.01)).

### 2.7. Evaluation of Potential Essential Genes in V. cincinnatiensis

**Figure 5:**
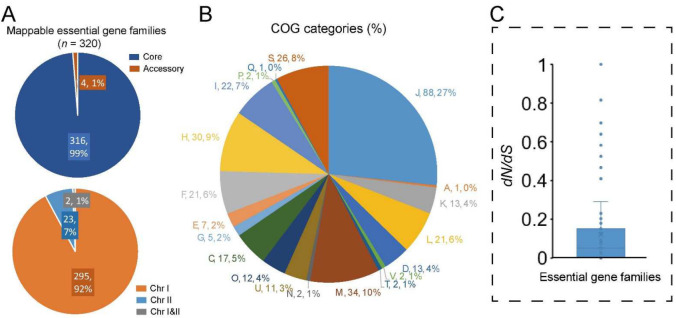
Analyses of the potential essential gene families in *V. cincinnatiensis:* (**A**) distribution of the potential essential gene families in pan-genome and chromosome location; (**B**) distribution of COG functional categories for the potential essential gene families; (**C**) the *dN*/*dS* rates of the potential essential gene families.

Essential genes play key roles in critical biological processes and their disruption will lead to the non-viability of the organism [31]. Previous studies have reported the essential genes in several *Vibrio* species [32,33,34]. Here, we identified the potential essential genes in *V. cincinnatiensis* through a comparison of the *V. cholerae* essential gene set (including 343 essential genes and 91 essential domains) identified by Chao et al. using high-density transposon mutagenesis [34]. A total of 320 (73.7%) of the *V. cholerae* essential genes have homologs in the *V. cincinnatiensis* pan-genome. Among these, 316 (98.8%) represented the core gene families and the remaining only four (1.2%) represented the accessory gene families (Figure 5A). Most of the essential gene families *(n* = 295; 91.6%) were located on Chr I; only 23 (7.2%) essential gene families were present on Chr II; two gene families were shared by Chr I and Chr II (Figure 5A).

Based on the COG annotation, essential gene families were significantly involved in conserved multicomponent pathways required for fundamental metabolic and structural functions (Figure 5B), including “J: translation, ribosomal structure, and biogenesis”, “D: cell cycle control, cell division, and chromosome partitioning”, “M: cell wall/membrane/envelope biogenesis”, “F: nucleotide transport and metabolism”, “H: coenzyme transport and metabolism”, and “I: lipid transport and metabolism” (Fisher’s exact test *p* value < 0.01). The *dN*/*dS* rates were estimated to investigate the conservation and evolutionary pressure for each essential gene family. Typically, the whole set of essential gene families was confirmed to have undergone significantly stronger evolutionary constraints (average *dN*/*dS* = 0.1 ± 0.2) than the whole core gene families (average *dN*/*dS* = 0.2 ± 0.3) (*t*-test, *p* = 0.02) (Figure 5C). Interestingly, the gene family that encoded DNA ligase (NAD(+)) (EC 6.5.1.2) was determinated to have undergone positive selection (*dN*/*dS* = 1.3). DNA ligase that catalyzes the formation of phosphodiester linkages using NAD as a coenzyme and as the energy source is essential for DNA replication and repair of damaged DNA [35]. Hence, the potential functional change caused by the mutation of DNA ligase sequences in *V. cincinnatiensis* deserves further attention.

### 2.8. Identification of Potential Horizontal Gene Families in V. cincinnatiensis

**Figure 6:**
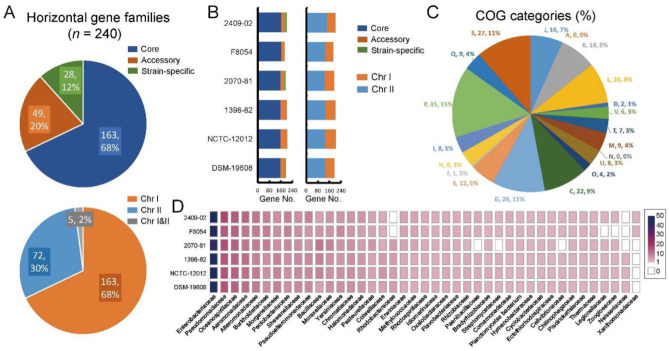
Analyses of the potential horizontal gene families in *V. cincinnatiensis:* (**A**) distribution of the potential horizontal gene families in pan-genome and chromosome location; (**B**) distribution and composition of the horizontal genes in *V. cincinnatiensis* genomes; (**C**) distribution of COG functional categories for the potential horizontal gene families; (**D**) the potential donor bacterial taxa providing donor genes for HGT.

HGT is the major driver of bacterial genomic evolution and genetic diversity [26]. Here, we explored the potential horizontal genes in *V. cincinnatiensis* genomes and quantified their composition and chromosome location. We identified 240 potential horizontal gene families, which included 163 core gene families (67.9%), 49 accessory gene families (20.4%), and 28 strain-specific genes (11.7%) (Figure 6A). On average, one genome harbored 196.5 ± 6.3 horizontal genes consisting of 160.2 ± 1.8 core genes, 31.7 ± 9.6 accessory genes, and 4.7 ± 5.6 strain-specific genes (Figure 6B). This result indicated that HGT contributed to the core genome, which conferred to *V. cincinnatiensis* species-specific properties during process of speciation. Most of the potential horizontal gene families (*n* = 163; 67.9%) were located on Chr I; only 72 (30.0%) gene families were present on Chr II; five gene families (2.1%) were shared by Chr I and Chr II (Figure 6A). A genome harbored an average of 133.8 ± 6.2 horizontal genes in Chr I and 62.7 ± 7.1 horizontal genes in Chr II (Figure 6B).

Based on the COG annotation, these horizontal gene families were significantly involved in several metabolism categories, including “C: energy production and conversion”, “G: carbohydrate transport and metabolism”, “P: inorganic ion transport and metabolism”, and “Q: secondary metabolites biosynthesis, transport, and catabolism”) (Fisher’s exact test *p* value < 0.05) (Figure 6C). It can be inferred that the acquisition of the novel metabolism-related genetic properties driven by HGT promoted the adaptation of *V. cincinnatiensis* into diverse niches. Furthermore, a total of 41 potential donor bacterial families were identified (Figure 6D). *Enterobacteriaceae* (46.3 ± 3.2), *Pseudoalteromonadaceae* (15.3 ± 1.03), *Oceanospirillaceae* (13.2 ± 0.4), *Aeromonadaceae* (10.8 ± 0.8), and *Alteromonadaceae* (9.0 ± 0.6) appeared to be the most common donors, indicating that *V. cincinnatiensis* might share some properties with these families.

### 2.9. Phylogenetic Position of V. Cincinnatiensis in the Genus Vibrio

**Figure 7:**
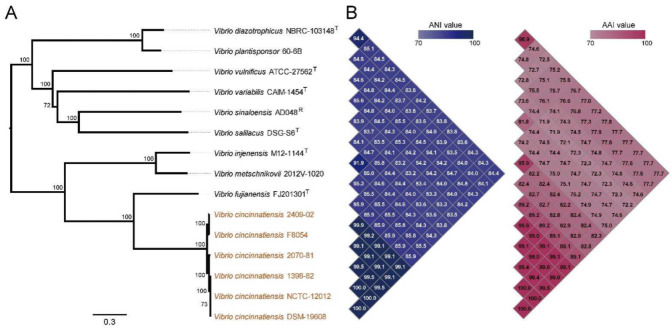
Phylogenetic, whole-genome nucleotide, and amino acid identity analysis. (**A**) Phylogenetic tree based on SNPs across 1673 single-copy core gene families shared by 6 *V. cincinnatiensis* genomes and nine reference genome of other *Vibrio* species was constructed by the ML method with 100 replicates; (**B**) the values next to the tree indicate average nucleotide identity (ANI) and average amino acid identity (AAI) values. The heatmap presents ANI (blue matrix) and AAI (red matrix) values.

To further explore the evolutionary dynamics of *V. cincinnatiensis*, we performed a phylogenetic analysis to determinate the evolutionary position of this species. Firstly, the reference 16S rRNA sequence (FUXB01000057) of *V. cincinnatiensis* was subjected to similarity-based searches against the taxonomically united 16S rRNA database in EzBioCloud [36] to identify similar species. All the 16S rRNA sequences (Table S5) of closely related species were collected from the EzBioCloud database and used to construct the maximum-likelihood (ML) tree (Figure S1). Secondly, nine reference genomes of the closest-related *Vibrio* species on the 16S tree (Figure S1) were collected in combination with six *V. cincinnatiensis* genomes for subsequent analyses. A high-resolution phylogeny based on 1673 single-copy core gene families that shared all 15 genomes was generated (Figure 7A). In the core genome tree, all *V. cincinnatiensis* members formed a monophyletic clade with a long branch length and were deeply nested within *Vibrio*, suggesting evolutionary divergence occurred between *V. cincinnatiensis* and other members of *Vibrio*. The nearest species to *V. cincinnatiensis* is *V. fujianensis* (FJ201301^T^), and they together with *V. metschnikovii* (2012V-1020) and *V. injenensis* (M12-1144^T^) formed a main clade.

The average nucleotide identity (ANI) and average amino acid identity (AAI) values were calculated to measure the genetic relatedness between *V. cincinnatiensis* and other members of *Vibrio* (Figure 7B). The strains shared high ANI (>99.1%) and AAI (>99.1%) values between each other. The ANI and AAI values determined from comparisons between *V. cincinnatiensis* and other members of *Vibrio* were 83.3–85.9% and 72.2–89.1%, respectively, which were lower than the 95% threshold value for species [37].

### 2.10. HGT Promoted Genotypic Profile of Virulence in V. cincinnatiensis

**Figure 8:**
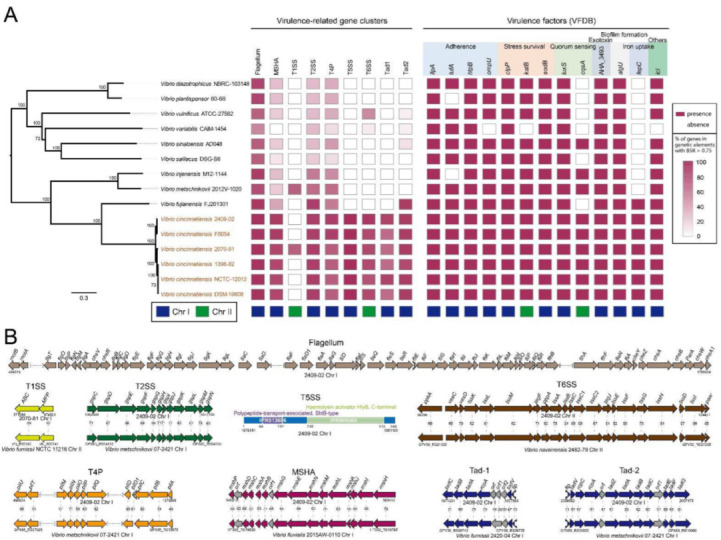
The genotypic profiles of virulence-related elements across six *V. cincinnatiensis* genomes. (**A**) Distribution of virulence-related elements. Color coding for virulence gene is based on the BLASTp parameters (E-value cutoff value of <1 × 10^−6^, identity value of >60%, and coverage value of >60%) calculated when the genomic data were screened against the reference sequences of VFDB database. Color coding for the gene clusters is based on the percentage of genes on a cluster that are present in a genome (defined as the BLASTp parameters of query gene with E-value cutoff value of <1 × 10^−6^, identity value of >60%, and coverage value of >60%); (**B**) the genetic organization of virulence-related elements. Homologous genes are shown in the same color and linked by dotted lines. The percentages of protein identities of homologous genes are shown.

Several pathogenic phenotypes of *V. cincinnatiensis* have been reported. To evaluate the potential pathogenicity, we identified the virulence-related elements, including fimbrial operon, secretion system operon, and virulence genes (Tables S6 and S7). A total of nine gene clusters and 13 genes, homologous to virulence-related elements identified in other pathogens, were identified in the *V. cincinnatiensis* genomes. These gene clusters were involved in encoding flagella, mannose-sensitive hemagglutinin (MSHA) pilus; type I (T1SS), II (T2SS), V (T5SS), and VI (T6SS) secretion systems; type IV pilus (T4P); and two types of Tad pili. The scattered virulence genes were associated with adherence (*ilpA*, *tufA*, *htpB*, and *ompU*), stress survival (*clpP*, *katB*, and *sodB*), quorum sensing (*luxS* and *cqsA*), exotoxin (AHA_3493), biofilm formation (*algU*), iron uptake (*fepC*), and others (*icl*). The distribution of these elements in all 15 *Vibrio* genomes is shown in Figure 8A. Most of these identified virulence-related elements are present in all six *V. cincinnatiensis* genomes except T1SS, which indicates that these virulence-related genotypic profiles represent a general property for *V. cincinnatiensis*. Parts of elements are absent in other related *Vibrio* genomes, such as MSHA, T1SS, T5SS, T6SS, Tad-1 and Tad-2 operon, *cqsA*, and *fepC*, suggesting that this is the result of HGT events. A tBLASTn search of the NCBI nucleotide collection (nt) databases using these elements showed that the majority of the highest homologs were identified in other *Vibrio* species, such as *V. furnissii* (harboring the highest homologs of T1SS and Tad-1), *V. navarrensis* (harboring the highest homolog of T6SS), and *V. fluvialis* (harboring the highest homolog of MSHA) (Figure 8B). Our results highlight that the potential HGT events from other *Vibrio* species promoted the pathogenicity of *V. cincinnatiensis*. Interestingly, although higher variability was exhibited by Chr II, most virulence-related elements were located on Chr I, except for T1SS operon, T6SS operon, *katB*, and *cqsA* (Figure 8A), indicating that Chr I plays an important role in the pathogenicity of *V. cincinnatiensis*.

The flagellum, MSHA pilus, T4P, T2SS, Tad-1 pilus, and Tad-2 pilus have been reported to contribute to the virulence of pathogenic *Vibrio* through motility, adhesion, or biofilm formation [38,39,40,41,42]. Furthermore, Sikora et al. reported that T2SS could confer fitness of *V. cholerae* in different ecological niches via the secretion of multiple proteins, including chitinases, proteases, DNase, and pilin [43]. T1SS operon was present only in one *V. cincinnatiensis* genome (2070-81), and T1SS could secrete multiple proteins involved in pathogenesis, nutrient acquisition, and antibacterial activity [44]. Interestingly, we did not identify any homologs with high identity (>40.0%) of the *V. cincinnatiensis* T5SS. Sequence analysis showed that this novel T5SS contained 549 amino acids, encoding one polypeptide-transport-associated ShlB-type domain (IPR013686) and one haemolysin activator HlyB C-terminal domain (IPR005565) (Figure 8B). The biological function of this T5SS deserves further research. T6SS in *Vbrio* has been reported to be associated with antimicrobial properties, antieukaryotic activities, and pathoadaptive fashion [45,46]. Finally, the identified genotypic profile of virulence highlighted the potential of *V. cincinnatiensis*. Future studies are required to confirm the function of these virulence-related elements in *V. cincinnatiensis* and their potential role in pathogenicity. In addition, *V. cincinnatiensis* has been reported to be associated with antimicrobial resistance and harbors several resistance-related genes (e.g., *sul1*, *sul2*, *aadA2*, and the class 1 integron) [8]. In this work, we did not mine any resistance genes in all six *V. cincinnatiensis* genomes.

## 3. Materials and Methods

### 3.1. Genome Collection and Annotation

All six *V. cincinnatiensis* genomes were collected and downloaded from NCBI GenBank database; nine other genomes were selected as representatives of *Vibrio* species related to *V. cincinnatiensis*. The estimates for genome completeness and contamination were performed using CheckM [47]. Gene prediction and annotation were performed using a consensus approach based on RAST server [48]. A detailed account of the genome dataset, including accession No., strain names, assembly type, genomic characteristics, completeness, and contamination is presented in Table S1. The scaffolds and contigs in draft genomes (NCTC-12012 and DSM-19608) were aligned to the chromosomes of the reference complete genome (2409-02) using Mauve Genome Alignment software 2.4.0 [49]. The scaffolds or contigs were mapped to the reference genome to determine their locations (Chr I or Chr II), with a cutoff of 60% coverage. All scaffolds in NCTC-12012 were mapped to the chromosome location. Only Contigs with 1372 (0.04%) bp length in DSM-19608 were unidentifiable.

### 3.2. Pan-Genome Analysis

Orthologous groups of protein-coding gene families of pan-genome were delimited using OrthoFinder 2.0 software [50,51]. The sequence files deposited in Orthogroup_Sequences folder were used to extract pan-genome families (the totality of all gene families found in *V. cincinnatiensis*), core genome families (genes shared among six genomes), accessory genome families (genes shared among more than one genome, but not in all), and strain-specific genes (genes found only in one genome). Curve fitting of the pan-genome was performed using a power-law regression based on Heap’s law (*n* = $\kappa N^{\kappa }$) [23,52], where *N* is the number of genomes, κ is a proportionality constant, and the growth exponent *γ* > 0 indicates an open pan-genome. A descriptive statistical analysis was generated using OriginPro 9 software with Allometric1 model. The gene families of the pan-genome were functionally characterized by the COG functional category [53] using eggNOG-mapper 1.0 software [54].

### 3.3. Identification of MGEs and Barriers to HGT

The tRNA loci were collected from the RAST annotation files [48]. The prophages were predicted using the online interface of phage search tool—enhanced release (PHASTER) [55]. The online interface of IslandViewer 4 [56] (integrated three different methods: SIGI-HMM [57], IslandPath-DIMOB [58], and IslandPick [59]) was utilized to identify the genomic islands. Insertion sequences were predicted using the online interface of ISfinder [60]. The TA systems were identified by BLASTp search of the dataset from TADB 2.0 database [61]. The RM systems were identified using the online interface of Restriction-ModificationFinder 1.1 [62]. The CRISPRs were predicted using the CRISPRCasFinder 4.2.2 software with default parameters [63].

### 3.4. Selective Pressure Analysis

Selective pressure in core gene families was estimated by calculating the ratio of the nonsynonymous substitution rate to the *dN*/*dS* rate. ParaAT software was used to codon-based align the orthologous sequences [64]. Then, the fast unconstrained Bayesian approximation (FUBAR) pipeline [65] within HYPHY software was used to measure the *dN*/*dS* ratio at each site in each core gene family.

### 3.5. Identification of Potential Horizontal Genes

HGTector software was used to identify the potential horizontal genes in *V. cincinnatiensis* species [66]. The *V. cincinnatiensis* (rank: species; taxon ID: 675) and *Vibrionaceae* (rank: family; taxon ID: 641) were set as self-group and close-group, respectively.

### 3.6. Phylogenetic Analysis

The core genome phylogenetic analysis was performed based on SNPs across single-copy core gene families extracted from the sequence files deposited in Orthogroup_Sequences folder. Nucleotide sequences of the single-copy core gene families were extracted according to the protein accession numbers and then aligned using the MAFFT 1.0 software [67]. The set of single-nucleotide polymorphisms (SNPs) presented in single-copy core gene families was extracted and then integrated according to the arrangement of the genes on the 2409-02 genome (complete genome). To avoid phylogenetic confusion, we identified and removed the putative recombinational regions from the SNPs set using ClonalFrameML 1.0 software [68]. The maximum likelihood (ML) tree was constructed using MEGA 7 [69] with the general time-reversible (GTR) model and 100 bootstrap replicates.

### 3.7. Genomic Characteristic Analysis

The JSpecies 1.2.1 software based on MUMmer method (ANIm) [37,70] and CompareM 2.0 software (https://github.com/dparks1134/CompareM, accessed on 30 January 2022) were used to calculate the average nucleotide identity (ANI) and amino acid identity (AAI), respectively.

### 3.8. Identification of Virulence-Related Genetic Elements and Resistance Genes

The detection and visualization of Macromolecular systems were performed using the programs MacSyFinder [71] and TXSScan [72] within Galaxy workflow system (https://galaxy.pasteur.fr/) (accessed on 30 January 2022) on the default parameters. To identify the virulence factors, the protein sequences of all genomes were aligned using BLASTp with an E-value cutoff < ×10^−6^, identity > 60%, and coverage > 60% against the dataset from the Virulence Factors Database (VFDB). The detection of the homologs of virulence-related elements was performed by tBLASTn against the NCBI Nucleotide collection (nt) databases. The domains of T5SS were predicted by InterPro annotation [73]. The resistance genes were predicted using ResFinder 4.0 software [74].

## 4. Conclusions

*V. cincinnatiensis* was found to distribute in aquatic environments worldwide. This poorly understood species is regarded as a potential human pathogen. In this work, we constructed a comprehensive comparative genomic analysis with six *V. cincinnatiensis* genomes to investigate the genetic diversity, genomic plasticity, evolutionary dynamics, phylogenetic position, and underlying mechanisms of pathogenicity of *V. cincinnatiensis*. The pan-genome exhibited genetic diversity with a flexible gene repertoire. The Chr II pan-genome harbored extensive genomic variability, about 45% of the pan-genome was variable. The genomic plasticity of *V. cincinnatiensis* was exhibited by tRNA loci and multiple types of MGEs, including GIs, prophages, and ISs. Meanwhile, the genomic stability was determinated by the barriers to HGT, including RM systems, TA systems, and CRISPR systems. Most HGT-related elements were heterogeneously distributed in two chromosomes. Comparative analysis revealed a significant difference in the functional enrichment between different components of pan-genome and chromosomes. The Chr I and Chr II core genomes mainly underwent a similar degree of evolutionary constraints of purifying selection. The potential essential genes in *V. cincinnatiensis* were mostly in the core gene families on Chr I and experienced significantly stronger evolutionary constraints than the whole core gene families. The potential horizontal HGT appears to have been an important evolutionary driver of genetic diversity, shaping the *V. cincinnatiensis* genomic evolution. Our phylogenetic analysis revealed that *V. cincinnatiensis* was closely related to *V. fujianensis*, *V. metschnikovii*, and *V. injenensis*. Our results also identified diverse virulence-related profiles in *V. cincinnatiensis*, including fimbrial operon, multiple pilus operons (encoding MSHA pilus, type IV pilus, and two types of Tad pili), secretion system operons (T1SS, T2SS, T5SS, and T6SS), and several scattered virulence genes. Most of them were located on Chr I. The distribution of these virulence-related elements in other related members of *Vibrio* species indicated that some of them seem to be the result of HGT events. Thus, HGT might play a key role in the pathogenicity of *V. cincinnatiensis*. It is important to note that that our conclusion is limited by the low number of *V. cincinnatiensis* genomes available included in the comparative genomic analyses. Furthermore, some potential informations especially for MGEs are missed from draft genomes (NCTC-12012 and DSM-19608). Thus, in favor of a more comprehensive analysis, more complete genomes, which represent an even distribution of *V. cincinnatiensis* genomic diversity, would be required in the future work.

## Figures and Tables

**Table 1:** General characteristics of the six genomes of *V. cincinnatiensis* included in this study.

Strain	Assembly	Genome			Chromosome I			Chromosome II		
		Length (bp)	GC Content (%)	Gene No.	Length (bp)	GC Content (%)	Gene No.	Length (bp)	GC Content (%)	Gene No.
2409-02	Complete	3,799,536	43.8	3533	2,796,027	44.2	2575	1,003,509	42.6	958
F8054	Complete	3,802,622	43.7	3533	2,768,337	44.2	2539	1,034,285	42.5	994
2070-81	Complete	3,807,910	43.8	3519	2,805,763	44.2	2591	1,002,147	42.6	928
1398-82	Complete	3,781,792	43.8	3496	2,771,467	44.2	2548	1,010,325	42.7	948
NCTC-12012	Scaffold	3,800,816	43.8	3541	2,772,819	44.2	2546	1,027,997	42.7	995
DSM-19608	Contig	3,665,830	43.7	3434	2,698,098	44.04	2525	966,360	42.8	908

## Data Availability

Not applicable.

## Supplementary Materials

The following supporting information can be downloaded at: https://www.mdpi.com/article/10.3390/ijms23094520/s1.
