# Supplementary material for: Comparative Genomic Analysis of Vibrio cincinnatiensis Provides Insights into Genetic Diversity, Evolutionary Dynamics, and Pathogenic Traits of the Species
Source: Int J Mol Sci. 2022 Apr 20;23(9):4520. doi: 10.3390/ijms23094520 (PMC9101195; doi:10.3390/ijms23094520)
Supplement: Supplementary file 1 [file ijms-23-04520-s001.zip › Supplementary legends.pdf]

International Journal of *Molecular Sciences*

MDPI

Supplementary Figure legends

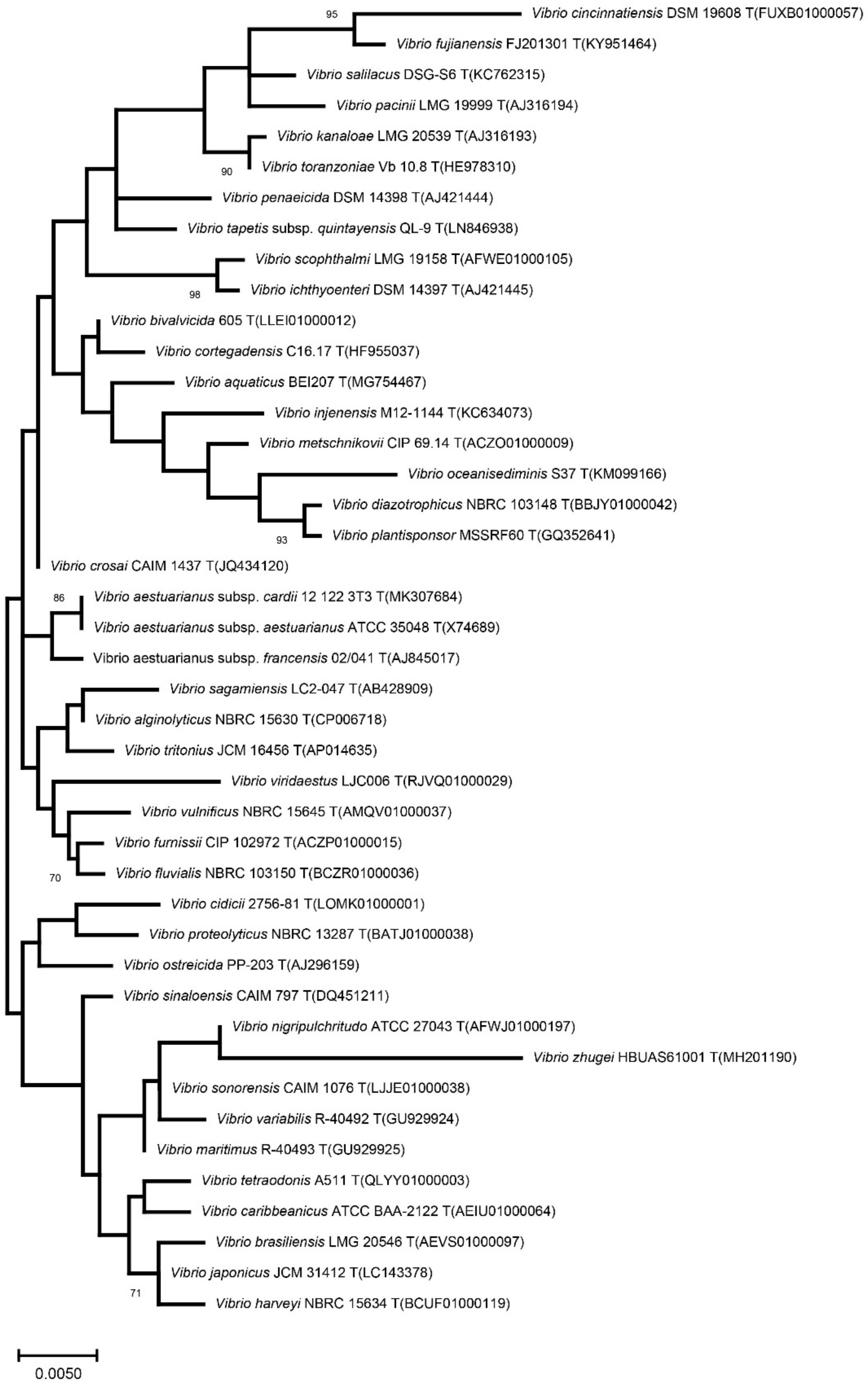

Supplementary Figure S1. Phylogenetic tree based on 16S rRNA sequences obtained by ML method with 1000 replicates. Numbers at nodes indicate the levels of bootstrap support (>70%).

Supplementary Table legends

Supplementary Table S1. List of public genome sequences in this study obtained from the GenBank database in NCBI.

Supplementary Table S2. List of the pan-genome of *V. cincinnatiensis*, including core, accessory, and strain-specific gene.

Supplementary Table S3. List of MGEs and barriers to HGT in the *V. cincinnatiensis* genomes.

Supplementary Table S4. List of the positively selected gene families ($dN/dS > 1$).

Supplementary Table S5. List of 16S rRNA sequences used in Fig. S1.

Supplementary Table S6. List of virulence-related elements in *V. cincinnatiensis*.

Supplementary Table S7. List of the virulence-related genes in *V. cincinnatiensis*.
