# Supplementary figures and images for: Comparative Genomic Analysis of Vibrio cincinnatiensis Provides Insights into Genetic Diversity, Evolutionary Dynamics, and Pathogenic Traits of the Species
Source: Int J Mol Sci. 2022 Apr 20;23(9):4520. doi: 10.3390/ijms23094520 (PMC9101195; doi:10.3390/ijms23094520)

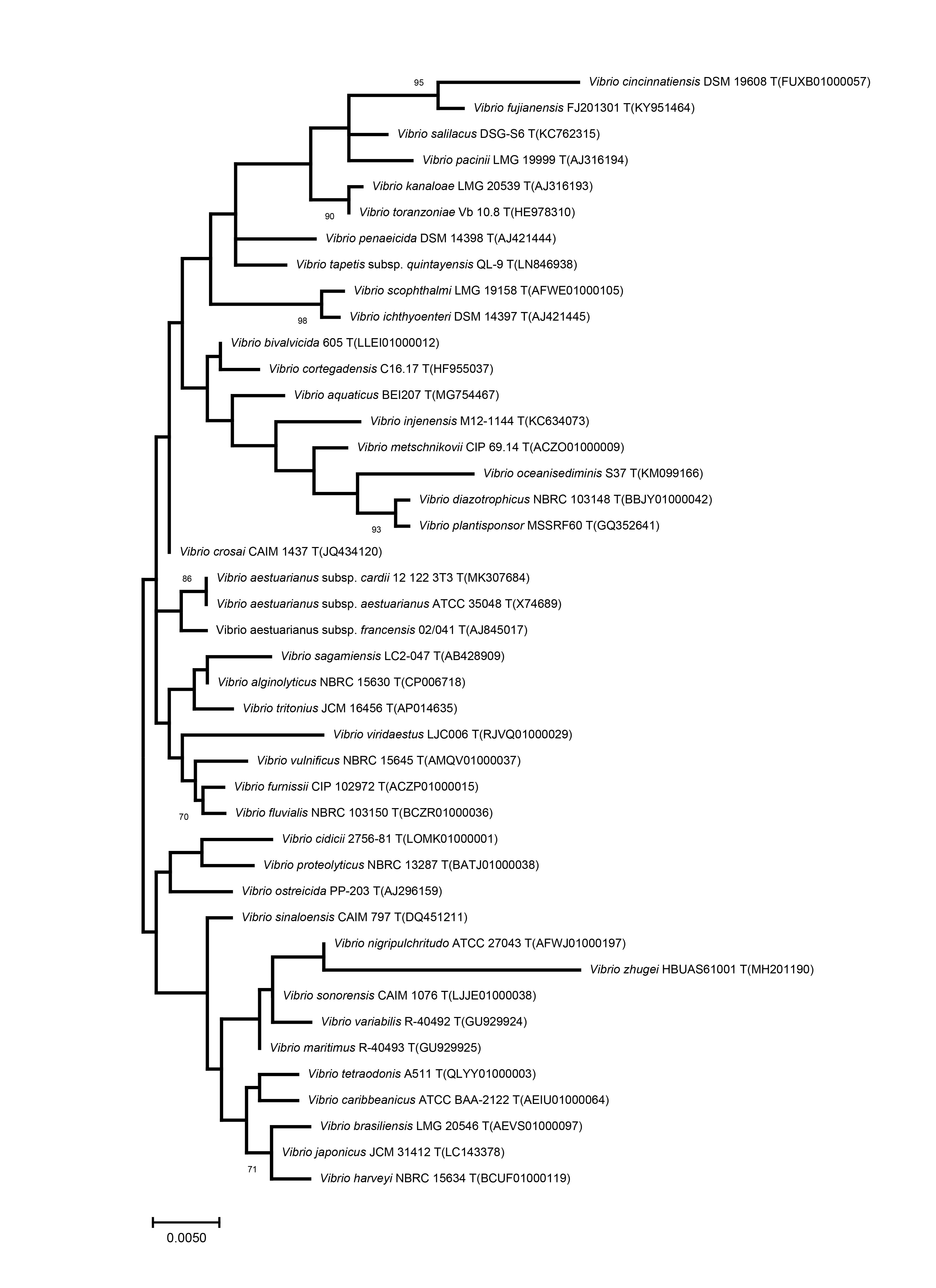

Supplement: Supplementary file 1 [file ijms-23-04520-s001.zip › Figure-S1.jpg]
